# Associations of Serum Substituted *p*-Phenylenediamine-Derived Quinone Levels with Oxidative Stress and Immune Function Biomarkers in a General Adult Population

**DOI:** 10.3390/toxics14080703

**Published:** 2026-08-09

**Authors:** Dandan Zhou, Lingzhen Pan, Xingxing Wu, Taoxiang Wang, Worou Chabi Noel, Desire Wade Atchike, Weili Mao, Danxia Zhao, Jianqiang Zhu

**Affiliations:** 1 Zhejiang Key Laboratory for Restoration of Damaged Coastal Ecosystems, Taizhou Environmental Science Design and Research Institute Co., Ltd., Taizhou University, Taizhou 318000, China; jinhanlin2000@zju.edu.cn (D.Z.); 21414082@zju.edu.cn (X.W.); wd8536378@163.com (T.W.); 2 Taizhou Jinghe Testing Technology Co., Ltd., Taizhou 318000, China; woshigunge@163.com; 3 Water Science and Technology Laboratory, National Water Institute, University of Abomey-Calavi, Abomey-Calavi P.O. Box 01 BP 526, Benin; woroucccy@yahoo.fr (W.C.N.); adesire@yahoo.fr (D.W.A.); 4 Department of Pharmacy, Quzhou Affiliated Hospital of Wenzhou Medical University, Quzhou People’s Hospital, Quzhou 324000, China; 5 Institute of Environmental Engineering Technology, School of Life Sciences, Taizhou University, Taizhou 318000, China

**Keywords:** 6PPD-Q, Human serum, Oxidative stress, MDA, Immune function

## Abstract

Substituted *p*-phenylenediamine-quinones (PPD-Qs) are commonly employed as antioxidants in varying rubbers with potential biotoxicological implications in humans. Associations between human PPD-Q exposure and oxidative stress and immune system modulation remain insufficiently characterized. This study investigated the human exposure profile of six PPD-Q homologues and their associations with malondialdehyde (MDA) and immune function biomarkers in a general adult population from Quzhou, China. The six target PPD-Qs were detected in most human serum samples (*n* = 205), with 2-((4-methylpentan-2-yl)amino)-5-(phenylamino)cyclohexa-2,5-diene-1,4-dione (6PPD-Q) emerging as the dominant compound (mean 1.67 ng/mL; range < LOD–5.46 ng/mL). Serum MDA levels showed significant positive correlations with several PPD-Qs, such as 6PPD-Q, 2,5-bis(o-tolylamino)cyclohexa-2,5-diene-1,4-dione (DTPD-Q), and 2,5-bis(phenylamino)cyclohexa-2,5-diene-1,4-dione (DPPD-Q), suggesting enhanced oxidative stress in humans. Moreover, the serum C-reactive protein level was positively associated with DTPD-Q, 77PD-Q (2,5-bis((5-methylhexan-2-yl)amino)cyclohexa-2,5-diene-1,4-dione), and DPPD-Q; IgA was negatively associated with CPPD-Q (2-(cyclohexylamino)-5-(phenylamino)cyclohexa-2,5-diene-1,4-dione) and 6PPD-Q; IgM was negatively associated with DPPD-Q and 6PPD-Q. These findings provide the first human correlational evidence suggesting associations between PPD-Q exposure and oxidative stress- and immune-related biomarkers, highlighting the need for further research and regulatory attention regarding these tire-derived pollutants.

## 1. Introduction

In recent years, the increasing demand for electric vehicles and consequent expansion in tire production underscores the need for improved tire durability [1,2,3]. To inhibit crack formation and thermal oxidation, substituted *p*-phenylenediamine antioxidants (PPDs) are extensively used in various rubber products, including tires for both conventional and electric vehicles [4,5]. Furthermore, manufacturers also employ PPDs as antioxidants across numerous rubber-based applications, such as sporting goods, recreational infrastructure, hoses, belts, and electrical cables [6,7]. Global PPD production has expanded significantly in response to substantial market demand [8,9]. China’s annual output alone grew from 100,000 metric tons in 2009 to >200,000 metric tons by 2020 [10]. This ubiquitous application results in the substantial environmental release of PPDs through multiple pathways [11,12]. Once discharged, multiple PPDs undergo complex transformation processes within environmental matrices, generating environmentally persistent PPD-quinones (PPD-Qs) [12,13].

Studies have observed widespread PPD-Q presence across various environmental matrices, including surface and groundwater systems, sediment, indoor dust, aquatic organisms, atmospheric samples, and potable water supplies [14]. Additionally, PPD-Qs in human biological fluids (namely urine, blood, and cerebrospinal fluid) has been documented [15,16,17]. This evidence underscores their pervasive human exposure and potential for human bioaccumulation [13]. The frequently detected PPD-Qs in environmental matrices and humans primarily include 6PPD-Q (2-((4-methylpentan-2-yl)amino)-5-(phenylamino)cyclohexa-2,5-diene-1,4-dione), IPPD-Q (2-(isopropylamino)-5-(phenylamino)cyclohexa-2,5-diene-1,4-dione), CPPD-Q (2-(cyclohexylamino)-5-(phenylamino)cyclohexa-2,5-diene-1,4-dione), and DPPD-Q (2,5-bis(phenylamino)cyclohexa-2,5-diene-1,4-dione) [13,18].

Growing concerns regarding PPD-Qs primarily arise from their demonstrated toxicological effects on aquatic organisms and humans, including immune modulation, hormone disruption, and carcinogenic risk [13,19]. Notably, 6PPD-Q exhibited significant neurotoxicity and had been implicated as a possible contributor to Parkinson’s disease pathogenesis [20,21]. Epidemiological evidence further linked 6PPD-Q exposure to developmental impairments, including growth retardation and compromised immune function in children [21]. Although increasing toxicological data highlight these associations, the potential relationships between human exposure to PPD-Q and adverse health effects remain incompletely characterized. Until now, the mechanistic associations between human PPD-Q exposure and oxidative stress and immune system modulation remain insufficiently characterized.

The regulation of the immune system is critically influenced by oxidative stress, which activates immune cells and increases the secretion of pro-inflammatory cytokines [22,23]. Extensive research demonstrates that oxidative damage to cellular components can generate oxidation-specific epitopes, which are subsequently detected by pattern recognition receptors on innate immune cells, thereby establishing a direct mechanistic connection between oxidative stress and inflammatory responses [24,25]. Moreover, a well-established indicator of oxidative stress in living organisms is malondialdehyde (MDA), which is generated as a byproduct of lipid peroxidation mediated by reactive oxygen [26,27]. C-reactive protein (CRP) contributes to innate immunity by facilitating pathogen recognition through calcium-dependent ligand binding [28,29]. Concurrently, immunoglobulin isotypes, such as IgA (immunoglobulin A), IgG (immunoglobulin G), and IgM (immunoglobulin M), exhibit specialized immune functions [30,31]. Quantitative assessment of CRP alongside serum IgA, IgG, and IgM levels has been commonly employed as clinical biomarkers to evaluate immune competence and inflammatory status in humans [32,33].

This study analyzed healthy adult serum samples (*n* = 205) from Quzhou to quantify concentrations of six PPD-Q homologues. Additionally, levels of oxidative stress and immune function biomarkers, including MDA, CRP, IgA, IgG, and IgM, were measured in the collected human serum samples. Importantly, this work presents the first investigation into potential correlations of serum PPD-Q levels with these biomarkers, providing novel insights into their associations with oxidative stress and immune modulation in humans.

## 2. Materials and Methods

### 2.1. Standard Chemicals

IPPD-Q, DPPD-Q, CPPD-Q, 6PPD-Q, 77PD-Q (2,5-bis((5-methylhexan-2-yl)amino)cyclohexa-2,5-diene-1,4-dione), and DTPD-Q (2,5-bis(*o*-tolylamino)cyclohexa-2,5-diene-1,4-dione), each with a purity level of 98% or higher, were obtained from SCRSTANDARD (Shanghai, China), TCI (Tokyo, Japan), and Sigma (Beijing, China). 6PPD-Q-d5 was acquired from Anpu Technology (Shanghai, China). Additional details regarding these reference standards can be found in Table S1 of Supporting Information (SI).

### 2.2. Recruitment of Study Subjects and Serum Sample Preparation

The current study enrolled healthy volunteers from the general population living in Quzhou, China, during January−July, 2024. The following inclusion criteria were applied: (1) age ≥ 30 years and ≤70 years; (2) long-term residency in Quzhou city for at least two consecutive years; (3) willingness to provide a fasting venous blood sample; and (4) ability to complete the structured questionnaire and provide written informed consent. The following exclusion criteria were applied: (1) self-reported history of autoimmune disorders (e.g., systemic lupus erythematosus and rheumatoid arthritis); (2) chronic inflammatory diseases (e.g., inflammatory bowel disease and chronic hepatitis); (3) acute infections or febrile illness within the past month; (4) current or previous malignancies; (5) use of immunosuppressive or anti-inflammatory medications within the past three months; and (6) pregnancy or lactation. A total of 239 adults were initially approached for participation. Of these, 34 individuals were excluded based on the above criteria (autoimmune disorders, *n* = 6; chronic inflammatory diseases, *n* = 11; acute infections, *n* = 7; immunosuppressive therapy, *n* = 5; refusal to participate, *n* = 5). The remaining 205 participants (104 men and 101 women) with no known chronic health conditions were included in the final analysis. Comprehensive demographic data, acquired through structured questionnaires, are available in Table 1. Certified nursing staff carried out standardized in-person interviews to systematically collect sociodemographic characteristics from all study subjects. Prior to study inclusion, each participant provided documented informed consent. The experimental protocol received formal approval from Quzhou People’s Hospital (Ethics Approval Number: QZ202404-6).

Venous blood specimens (5 mL) were obtained from participants under fasting conditions in the morning using coagulation-activated vacutainer tubes (Becton Dickinson, Franklin Lakes, NJ, USA). After collection, blood samples were centrifuged (3800× *g*; 15 min) after clot. Resulting serum fraction samples were immediately stored at −60 °C. Five field blank controls, ultrapure water (*n* = 6), were treated identically to biological specimens, undergoing the same centrifugation, aliquoting, and storage protocols as the serum samples.

### 2.3. PPD-Q Analysis in Human Serum

Human serum was extracted, following prior methods [16]. During extraction procedures, 1.0 mL human serum was spiked with 5.0 ng of 6PPD-Q-d5, and 3 mL of MTBE was mixed to each serum, which was followed by rigorous vortex mixing for 15 min. Phase separation was achieved through centrifugation (4000× *g*; 10 min; 4 °C). Obtained MTBE layer was quantitatively transferred to a clean glass tube. To ensure maximum recovery, a secondary extraction was performed with another 3 mL portion of fresh MTBE solvent. The pooled organic phases were then concentrated to complete dryness using a gentle nitrogen at 25 °C. Acquired resulting residue was finally reconstituted with HPLC-grade methanol (50 μL) for instrumental analysis.

Chromatographic separation of PPD-Qs was achieved on a Hypersil C18 column (50 mm × 2.1 mm, 3 μm; Thermo-Scientific, Waltham, MA, USA). The analysis was performed using an ACQUITY UPLC system coupled to a Xevo TQ-S mass spectrometer (Waters Co., Ltd., Milford, MA, USA). A binary mobile phase composed of (A) acidified water (0.25% acetic acid, 5.0 mM ammonium acetate) and (B) methanol was delivered at 0.20 mL/min with the following gradient: 10% B from 0 to 0.5 min, ramped to 25% B by 1.0 min, increased to 95% B at 10 min (held for 3 min), then returned to 10% B in 0.1 min with a 4.0 min re-equilibration. PPD-Q detection was performed in positive electrospray ionization mode. The specific multiple reaction monitoring MRM transitions are detailed in Table S2 (SI).

Blank controls (pure water) were analyzed alongside human serum samples to assess potential PPD-Q contamination. Methanol solvent blanks and procedural blanks (1.0 mL water) were inserted and analyzed after every ten serum samples. Experimental materials were pre-screened to confirm the absence of detectable PPD-Qs. We quantified PPD-Q levels in human serum by internal standard calibration. Six-point matrix-matched calibration curves (0.50–100 ng/mL) were constructed for each PPD-Q homologue, demonstrating excellent linearity (R^2^ > 0.995). Limits of detection (LODs), determined at S/N of 3:1, ranged from 0.025 to 0.098 ng/mL for target PPD-Qs. Method accuracy was confirmed via spike-recovery tests, which were performed on human serum spiked at three concentrations. This experiment yielded extraction recoveries of 83–107% for all PPD-Q homologues. To assess method precision, human serum samples (*n* = 5) were spiked with the PPD-Q mixture at 1.0 ng/mL or 10 ng/mL, and then analyzed. The intra-day relative standard deviations (RSDs) were <10%, and the inter-day variability (assessed over seven days) was <15%. Full validation data (i.e., LODs and extraction recoveries of PPD-Qs) are provided in SI, Table S3.

### 2.4. Analysis of MDA and Immune Function Biomarkers in Human Serum

In this study, the selection of immune function markers was based on their well-established clinical relevance and analytical robustness. CRP is a widely recognized acute-phase reactant and a sensitive biomarker of systemic inflammation. Immunoglobulins (IgA, IgG, and IgM) represent key effectors of humoral immunity: IgA mediates mucosal defense, IgG constitutes the predominant antibody class in systemic circulation responsible for secondary immune responses, and IgM serves as the primary antibody produced during primary immune responses. These selected markers provide a practical and reliable foundation for the initial exploration of potential associations between PPD-Q exposure and immune function in humans.

Serum levels of MDA, CRP, IgM, IgA, and IgG in study subjects were quantitatively analyzed using standardized commercial kits, according to manufacturer protocols. In brief, serum MDA levels were assayed via the thiobarbituric acid reaction (TBARS; Allenda Biotech., Shanghai, China). Total serum IgA, IgG, and IgM concentrations were determined by immunoturbidimetry using Tina-quant IgA Gen.2, Tina-quant IgG Gen.2, and Tina-quant IgM Gen.2 assays, respectively, on a Roche/Hitachi cobas c501 automated clinical chemistry analyzer (Roche Diagnostics GmbH, Mannheim, Germany). CRP levels in human serum were assessed using latex-enhanced immunoturbidimetry (Saipei Biotech.; Wuhan, China). Detailed analyzing methods for MDA, CRP, IgA, IgM, and IgG in human serum are provided in SI.

### 2.5. Statistical Analysis

Normality of serum concentrations for PPD-Q homologues, MDA, and immune function biomarkers was checked using Shapiro–Wilk test. Concentration values below the LODs for PPD-Q analytes were substituted with LOD/√2, which represents a standard imputation approach for censored data. Calculation of mean serum concentrations was restricted to PPD-Q compounds exhibiting detection frequencies > 50%. Spearman’s rank-order correlation (ρ value) evaluated monotonic relationships between serum concentrations of different PPD-Q homologues in subjects. Comparative analyses of human serum concentration differences across PPD-Q homologues and gender-based concentration disparities employed Mann–Whitney *U* test. What is more, this study applied multiple linear regression (MLR) for quantifying relationships between ln-transformed human serum PPD-Q concentrations (natural log-transformation mitigated right-skewness) and five continuous serum biomarkers (i.e., MDA, IgA, IgM, IgG, and CRP). Each biomarker served as a separate dependent variable. The model incorporated a priori covariates with established biological relevance to immune regulation, such as age, sex, body mass index, education level (categorical), occupational status (categorical), marital status (categorical), residential location (urban/rural), alcohol intake frequency (categorical), and tobacco use status (categorical). Human PPD-Q exposure effects were expressed as beta coefficients (*β*) and their 95% confidence intervals (95% CIs). This study applied the Benjamini–Hochberg procedure to control the false discovery rate (FDR). An FDR-corrected *p* < 0.05 was considered statistically significant.

## 3. Results

### 3.1. Detection of Human Serum PPD-Qs

Female participants had a mean age of 46 ± 16 years, while males averaged 42 ± 14 years (*p* = 0.25, Table 1). Additionally, we found no significant distinctions (*p* > 0.05) in BMI, education level, occupational status, marital status, or residence between the male and female subjects. However, significant gender-based variations (*p* < 0.05) were observed in alcohol consumption (63% vs. 12%), spicy food intake (42% vs. 15%), and tobacco use (62% vs. 8.9%), with male participants consistently exhibiting higher rates than females.

**Figure 1:**
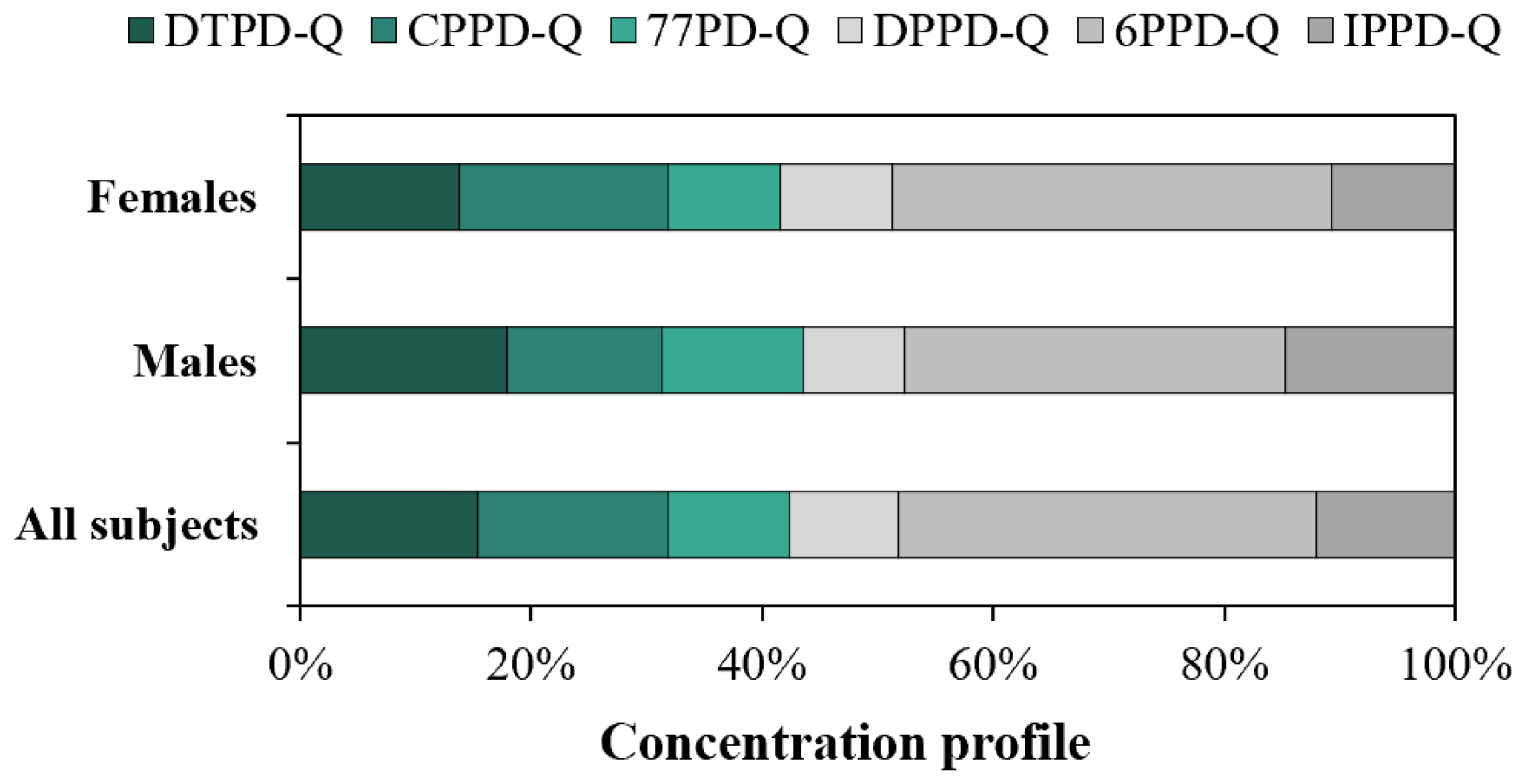
Concentration profiles of PPD-Qs in human serum from males, females, and all subjects.

Analysis of human serum samples revealed detectable levels of all target PPD-Qs, with detection frequencies ranging from 71% to 92% (Table 2). These findings indicate widespread exposure to PPD-Q homologues among the general Chinese adults. We found that 6PPD-Q was the most frequently detected PPD-Q, occurring in over 90% of human serum samples. Total PPD-Q concentrations (∑PPD-Qs) ranged from 0.13 to 16 ng/mL (mean: 4.7 ng/mL) in human serum, with 6PPD-Q accounting for 36% of the total PPD-Q concentration (Figure 1). DTPD-Q and CPPD-Q were the next most abundant PPD-Q congeners, with mean concentrations of 0.76 ng/mL and 0.75 ng/mL, respectively. Sex-based differences were noted for serum CPPD-Q levels, with female subjects showing significantly higher levels than males (0.89 vs. 0.61 ng/mL; *p* = 0.046). Correlation analysis (SI, Table S4) revealed significant positive associations between 6PPD-Q and CPPD-Q (ρ = 0.54, *p* < 0.01), 77PD-Q (ρ = 0.42, *p* = 0.028), and DPPD-Q (ρ = 0.53, *p* < 0.01) in human serum. Similarly, significant correlations were observed in human serum between IPPD-Q and DTPD-Q (ρ = 0.46, *p* = 0.040), 77PD-Q (ρ = 0.68, *p* < 0.01), and DPPD-Q (ρ = 0.68, *p* < 0.01).

The ubiquitous detection of PPD-Qs in participants’ serum raises important concerns regarding the potential exposure sources. In urban environments, traffic-related emissions are likely a primary contributor, as tire wear particles generated from road abrasion are a major source of PPD-Qs in ambient air, road dust, and runoff [6,7,9]. Individuals living or working in close proximity to major roadways or industrial zones may experience higher inhalation exposure to PPD-Q-laden particulate matter [9]. Additionally, indoor dust has also been identified as a significant PPD-Q exposure source, as PPD-Qs have been detected in household dust samples [6,7]. Furthermore, dietary intake and drinking water consumption may contribute to PPD-Q exposure [9,11].

### 3.2. Oxidative Stress and Immune Markers in Humans

**Figure 2:**
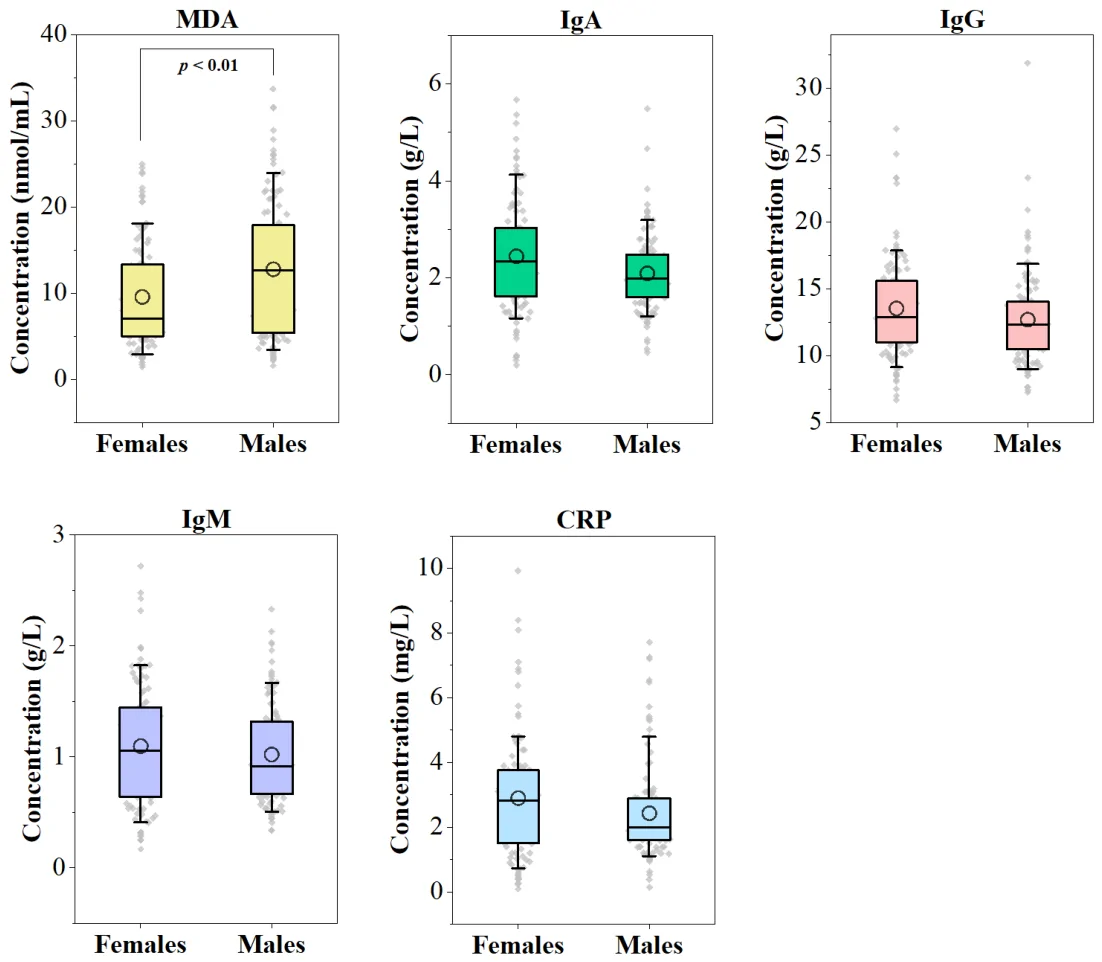
Concentrations of MDA, IgA, IgG, IgM, and CRP in human serum from female and male participants.

We observed a mean serum MDA level of 11.3 nmol/mL, with values ranging from 1.42 to 33.7 nmol/mL (Figure 2). The present study found that male participants had significantly higher serum MDA levels (12.9 nmol/mL) compared to females (9.71 nmol/mL), with a statistically significant difference (*p* < 0.01). Levels of MDA were positively (ρ = 0.71, *p* < 0.01) correlated with age in human serum. Specifically, mean human serum levels of MDA were gradually increased across age groups, displaying mean levels of 8.08 nmol/mL, 11.3 nmol/mL, 15.3 nmol/mL, and 16.6 nmol/mL in 30–40, 41–50, 51–60, and 61–70 age groups, respectively.

As shown in Figure 2, the measured mean ± SD serum concentrations were 2.36 ± 1.46 g/L, 13.2 ± 3.78 g/L, 1.04 ± 0.66 g/L, and 2.47 ± 1.71 mg/L for IgA, IgG, IgM, and CRP, respectively. Females had higher mean serum concentrations of IgA (2.52 g/L vs. 2.22 g/L), IgG (13.3 g/L vs. 12.7 g/L), IgM (1.18 g/L vs. 0.97 g/L), and CRP (2.66 mg/L vs. 2.19 mg/L) than males (Figure 2), but none of these differences were significant (*p* > 0.05). For comparison, the commonly adopted reference values in healthy adults are 0.70–4.00 g/L for IgA, 7.00–16.00 g/L for IgG, 0.40–2.30 g/L for IgM, and <5.0 mg/L for CRP.

### 3.3. Associations Between PPD-Q Exposure and Oxidative Stress

We observed a significant positive association (*p* = 0.025) between serum concentrations of ∑PPD-Qs and serum MDA levels (Table 3). We found that each one-unit increase in log-transformed DTPD-Q corresponded to a 0.35 increase in serum MDA (95% CI: 0.13, 0.60). Additionally, ln-transformed DPPD-Q (*β* = 0.16, 95% CI: 0.041, 0.28; *p* = 0.039) and 6PPD-Q (*β* = 0.24, 95% CI: 0.17, 0.31; *p* = 0.022) levels were significantly associated with higher MDA levels.

### 3.4. Associations Between PPD-Q Levels and Immune Interference

We observed that each one-unit increase in ln-transformed ∑PPD-Qs was associated with a 0.17 increase in serum CRP (95% CI: 0.054, 0.29; *p* < 0.01), a 0.22 increase in IgA (95% CI: 0.050, 0.41; *p* = 0.036), and a 0.27 increase in IgM (95% CI: 0.10, 0.45; *p* = 0.029) (Table 4). Focusing on individual congeners, we found that one-unit increases in ln-DTPD-Q, ln-77PD-Q, and ln-DPPD-Q were associated with respective increases of 0.45 (95% CI: 0.20, 0.63), 0.26 (95% CI: 0.12, 0.42), and 0.24 (95% CI: 0.12, 0.36) in ln-CRP. Conversely, ln-CPPD-Q (*β* = −0.28, 95% CI: −0.42, −0.14; *p* < 0.01) and ln-6PPD-Q (*β* = −0.35, 95% CI: −0.54, −0.16; *p* < 0.01) exhibited a negative correlation with IgA. For IgM, each ln-unit increase in DPPD-Q (*β* = −0.25, 95% CI: −0.38, −0.14; *p* = 0.033) and 6PPD-Q (*β* = −0.50, 95% CI: −0.69, −0.43; *p* < 0.01) corresponded to a decrease.

## 4. Discussion

In this cross-sectional investigation, we present, to our knowledge, the first descriptive epidemiological data revealing correlations between human serum PPD-Q concentrations and levels of oxidative stress and immune function biomarkers. These associations should be interpreted as hypothesis-generating and do not imply causation. We identified significant positive associations between elevated serum DTPD-Q, DPPD-Q, and 6PPD-Q concentrations and increased MDA levels. Furthermore, we found that increased exposure to several PPD-Qs was significantly associated, in either a positive or negative direction, with serum levels of CRP, IgA, IgM, and IgG.

Detection frequencies exceeding 70% were observed for all target PPD-Qs in subject serum samples, confirming widespread human exposure within the cohort. Among these analytes, 6PPD-Q emerged as the predominant homologue, followed by DTPD-Q and CPPD-Q. Current data on human serum PPD-Q concentrations remains limited. We found our results to be consistent with Han et al. (2025), who identified 6PPD-Q as the dominant PPD-Q in human serum from Anhui, China (*n* = 20; mean 1.4 ng/mL) [16]. However, they reported higher mean serum IPPD-Q (0.80 ng/mL) and 77PD-Q (0.688 ng/mL) than we observed, while their CPPD-Q (0.48 ng/mL) and DTPD-Q (0.56 ng/mL) concentrations were lower. Notably, the mean DTPD-Q level (0.76 ng/mL) was substantially higher than that reported in pregnant women from Jining (0.185 ng/mL; *n* = 65) by [34]. Peoples from Tianjin (< 0.05 ng/mL) [35] and Chongqing (0.84 ng/mL) [36] had higher mean 6PPD-Q concentrations than we measured. These differences across studies likely reflect geographic and environmental variability in exposure, as well as demographic factors such as age, diet, and lifestyle.

Serum MDA levels are commonly used to assess lipid peroxidation, indicating the extent of oxidative stress in humans [37,38]. We observed that, in human subjects, serum MDA concentrations were positively correlated with exposure to certain PPD-Qs. Although there are currently no direct reports of PPD-Q exposure inducing oxidative stress in humans, animal studies had provided relevant experimental evidence supporting this effect. Previous in-vivo studies had found that oxidative stress could be induced by 6PPD-Q in a variety of biota [13,39,40]. For example, the oxidative stress level was significantly elevated in the brain of mice after 6PPD-Q exposure [41]. Exposure to 6PPD-Q may induce intestinal damage in *Caenorhabditis elegans* through oxidative stress-mediated mechanisms [42]. Similarly, several PPD-Qs (such as DPPD-Q, CPPD-Q, and IPPD-Q) had been shown to trigger oxidative stress in the aquatic bacterium Vibrio fischeri [43]. This suggests a significant association between exposure to these PPD-Qs and oxidative damage in humans, potentially affecting various health outcomes.

A noteworthy finding of our study is the congener-specific association patterns observed between individual PPD-Qs and immune biomarkers. For instance, ∑PPD-Qs was positively associated with IgA, yet CPPD-Q and 6PPD-Q showed negative associations with this immunoglobulin, whereas IPPD-Q showed a positive association. Similarly, ∑PPD-Qs was positively associated with IgM, while both DPPD-Q and 6PPD-Q exhibited negative associations. These divergent patterns suggest that different PPD-Q congeners may possess distinct toxicological profiles and biological activities, rather than acting as a single homogeneous chemical class. This may be due to the structural differences among PPD-Q congeners, particularly the variations in alkyl side-chain length, branching, and aromatic substitution. These findings further highlight the importance of congener-specific risk assessment, rather than treating PPD-Qs as a single entity. Future toxicological and epidemiological studies are urgently needed to elucidate the structure-activity relationships and underlying mechanisms driving these congener-specific effects.

The immunotoxicity of PPD-Q compounds in humans has not been thoroughly characterized. Comprehensive epidemiological evidence linking PPD-Q exposure to immune dysfunction in humans is currently lacking. However, emerging toxicological evidence points to plausible mechanisms by which these compounds may disrupt immune homeostasis. For instance, Zhang et al. (2024) reported an association between elevated 6PPD-Q exposure from kindergarten dust and compromised immunity in children, manifesting as growth impairment [21]. He at al. demonstrated that repeated 6PPD-Q administration in male mice induced splenic atrophy, marked by histopathological alterations and reduced spleen index, which represents a hallmark of immunosuppression [44]. Furthermore, recent findings suggested that PPD-Qs may impair gut barrier integrity, promoting systemic inflammation and immune dysregulation [45]. Collectively, these studies imply the potential for PPD-Q exposure to adversely affect immune function in humans. In addition, concentrations of MDA were significantly correlated with some immune function biomarkers (i.e., CRP, IgA, and IgM; SI, Table S5) in human serum. This indicates a potential relationship between oxidative stress and immune response modulation. These findings are consistent with a hypothesized pathway in which PPD-Q exposure is associated with oxidative stress and correlated with alterations in immune function markers. However, due to the cross-sectional nature of our study, this proposed pathway remains speculative and requires confirmation through experimental and longitudinal investigations. Although these toxicological studies from animal models provide biological plausibility for our observed associations, it is important to emphasize that our cross-sectional human data cannot establish causality. The associations we report should be viewed as hypothesis-generating evidence that supports, but does not prove, a potential role of PPD-Qs in oxidative and immune disturbances.

We recognize several limitations in this study. First, owing to the cross-sectional design, we cannot draw causal conclusions regarding the relationship between PPD-Q exposure and changes in serum MDA or immune biomarkers. Future longitudinal or experimental investigations are needed to further confirm these findings. While widely employed in population-based biomonitoring studies due to its simplicity and cost-effectiveness, the TBARS assay is not entirely specific for MDA, potentially leading to an overestimation of MDA levels. Second, all serum samples were collected from a single urban population in eastern China (Quzhou City). We acknowledge that the single-center design inherently limits the generalizability of our findings to populations residing in regions with markedly different environmental pollution profiles, climatic conditions, and dietary or lifestyle patterns. Third, although multiple confounding variables were adjusted in the regression models, unmeasured confounders may still have influenced the results. Additionally, the human PPD-Q exposure was assessed only based on a single serum sample, which may not reflect long-term exposure. The lack of repeated measurements precludes a robust assessment of long-term PPD-Q exposure patterns. Future prospective cohort studies with repeated serum sampling are warranted to confirm our findings and to better characterize the chronic health effects of PPD-Q exposure. Lastly, while the study focused on several key humoral immune parameters, it did not assess cellular immune responses or other immune pathways that may also be affected by PPD-Q exposure. Future research should incorporate larger and more heterogeneous populations and include a broader array of immune and toxicological endpoints. Lastly, while our study focused on several key humoral immune parameters (CRP, IgA, IgG, and IgM), we acknowledge that cellular immune responses (e.g., T-lymphocyte subpopulations, natural killer cell activity) and inflammatory cytokines (e.g., interleukins such as IL-6, IL-10, TNF-α, and interferons) were not assessed. These markers represent critical components of the immune system and may provide complementary insights into the immunomodulatory effects of PPD-Q exposure. The evaluation of these cellular and cytokine-mediated pathways is warranted in future investigations to provide a more comprehensive immunotoxicology assessment of PPD-Qs in humans.

## 5. Conclusions

This study provides the first epidemiological evidence suggesting associations between human serum PPD-Q concentrations and biomarkers of oxidative stress and immune function in a general adult population. Serum concentrations of multiple PPD-Q congeners, including 6PPD-Q, DTPD-Q, and DPPD-Q, were significantly correlated with greater serum MDA levels. Furthermore, both positive and negative associations were observed between PPD-Q exposure and critical immunological indicators (e.g., CRP, IgA, IgM, and IgG). Given the widespread detection of serum PPD-Qs and their associations with key biomarkers in humans, our results underscore the urgent need for further longitudinal and mechanistic studies to evaluate health risks and inform future regulatory policies concerning human exposure to these tire-derived contaminants.

## Figures and Tables

**Table 1:** Demographic Profiles of male and female participants.

	Males (*n* = 104)		Females (*n* = 101)		*p*-Value
	*n*	Percentage	*n*	Percentage	
**Age (years)**					0.25
30–40	28	27%	34	34%	
41–50	29	28%	33	33%	
51–60	28	27%	20	20%	
61–70	19	18%	14	14%	
**Body mass index (kg/m^2^)**					0.17
<18.5	15	14%	22	22%	
18.5–24.5	76	73%	67	66%	
>24.5	13	13%	12	12%	
**Educational level**					0.28
Lower than high school	30	29%	38	38%	
High school	54	52%	49	49%	
College	20	19%	14	14%	
**Occupational status**					0.30
Employed	74	71%	70	69%	
Unemployed	30	29%	31	31%	
**Marital status**					0.44
Married	82	79%	79	78%	
Unmarried	22	21%	22	22%	
**Residence**					0.74
Urban region	77	74%	75	74%	
Rural region	27	26%	26	26%	
**Alcohol consumption**					<0.01
No	38	37%	89	88%	
Yes	66	63%	12	12%	
**Spicy food consumption**					0.029
No	60	58%	86	85%	
Yes	44	42%	15	15%	
**Tobacco smoking**					<0.01
No	40	38%	92	91%	
Yes	64	62%	9	8.9%	

**Table 2:** Concentrations (ng/mL) of PPD-Qs in human serum from participants (*n* = 205).

	Detection Frequency	Mean	Median	Concentration Percentile			
				Min	25th	75th	Max
***Females***							
**DTPD-Q**	81%	0.68	0.47	<LOD	0.13	0.78	3.74
**CPPD-Q**	81%	0.89	0.69	<LOD	0.32	1.26	3.37
**77PD-Q**	87%	0.48	0.30	<LOD	0.092	0.48	5.64
**DPPD-Q**	84%	0.48	0.19	<LOD	0.078	0.42	4.47
**6PPD-Q**	94%	1.87	1.44	<LOD	0.62	2.93	5.46
**IPPD-Q**	71%	0.53	0.23	<LOD	<LOD	0.61	3.24
***Males***							
**DTPD-Q**	86%	0.82	0.47	<LOD	0.21	1.17	3.85
**CPPD-Q**	84%	0.61	0.24	<LOD	0.075	0.88	3.26
**77PD-Q**	88%	0.56	0.44	<LOD	0.073	0.68	4.39
**DPPD-Q**	81%	0.40	0.13	<LOD	0.086	0.47	2.71
**6PPD-Q**	90%	1.51	0.72	<LOD	0.58	2.95	5.37
**IPPD-Q**	72%	0.67	0.38	<LOD	<LOD	0.88	3.11

**Table 3:** Beta coefficients (*β*) with 95% confidence interval (CI) for the relationship between serum MDA levels and Ln-transformed serum PPD-Q concentrations in the study cohort.

	*β*, (95% CI),	*p*-Value
**DTPD-Q**	0.35 (0.13, 0.60)	<0.01
**CPPD-Q**	0.15 (−0.14, 0.31)	0.28
**77PD-Q**	0.030 (−0.15, 0.18)	0.40
**DPPD-Q**	0.16 (0.041, 0.28)	0.039
**6PPD-Q**	0.24 (0.17, 0.31)	0.022
**IPPD-Q**	0.073 (−0.36, 0.41)	0.74
**∑PPD-Qs**	0.19 (0.094, 0.30)	0.025

**Table 4:** Beta coefficients (*β*) with 95% confidence interval (CI) for the relationship between serum immune-relative parameter levels and Ln-transformed serum PPD-Q concentrations in the study cohort.

	*β*, (95% CI)			
	CRP	IgA	IgG	IgM
**DTPD-Q**	0.45 (0.20, 0.63)	−0.064 (−0.29, 0.18)	0.30 (0.034, 0.67)	0.097 (−0.28, 0.35)
	*p* < 0.01	*p* = 0.59	*p* = 0.12	*p* = 0.69
**CPPD-Q**	0.12 (−0.045, 0.22)	−0.28 (−0.42, −0.14)	−0.15 (−0.29, −0.062)	0.024 (−0.11, 0.16)
	*p* = 0.44	*p* < 0.01	*p* = 0.066	*p* = 0.73
**77PD-Q**	0.26 (0.12, 0.42)	0.17 (0.065, 0.29)	−0.16 (−0.41, 0.11)	0.31 (0.074, 0.63)
	*p* = 0.045	*p* = 0.080	*p* = 0.40	*p* = 0.037
**DPPD-Q**	0.24 (0.12, 0.36)	0.089 (−0.19, 0.30)	0.17 (−0.032, 0.38)	−0.25 (−0.38, −0.14)
	*p* = 0.030	*p* = 0.29	*p* = 0.35	*p* = 0.033
**6PPD-Q**	0.16 (−0.083, 0.28)	−0.35 (−0.54, −0.16)	0.027 (−0.22, 0.29)	−0.50 (−0.69, −0.43)
	*p* = 0.46	*p* < 0.01	*p* = 0.50	*p* < 0.01
**IPPD-Q**	0.082 (0.18, 0.25)	0.27 (0.18, 0.31)	−0.10 (−0.45, 0.27)	0.093 (−0.21, 0.36)
	*p* = 0.28	*p* < 0.01	*p* = 0.20	*p* = 0.42
**∑PPD-Qs**	0.17 (0.054, 0.29)	0.22 (0.050, 0.41)	0.087 (−0.14, 0.20)	0.27 (0.10, 0.45)
	*p* < 0.01	*p* = 0.036	*p* = 0.29	*p* = 0.029

## Data Availability

The original contributions presented in this study are included in the article/Supplementary Materials. Further inquiries can be directed to the corresponding authors.

## Supplementary Materials

The following supporting information can be downloaded at https://www.mdpi.com/article/10.3390/toxics14080703/s1, Table S1: List of target PPD-Qs in the present study and their abbreviations and full names; Table S2: MRM transition parameters for PPD-Qs and internal standard; Table S3: LODs and extraction recoveries of PPD-Qs in human serum; Table S4: Correlations in human serum concentrations among different PPD-Qs; Table S5: Correlations between MDA and immune function biomarkers in human serum.

## Abbreviations

The following abbreviations are used in this manuscript:

PPD-Q	*p*-Phenylenediamine-quinone
MDA	Malondialdehyde
CRP	C-reactive protein
IgA	Immunoglobulin A
IgG	Immunoglobulin G
IgM	Immunoglobulin M
LOD	Limits of detection
