# Supplementary material for: Associations of Serum Substituted p-Phenylenediamine-Derived Quinone Levels with Oxidative Stress and Immune Function Biomarkers in a General Adult Population
Source: Toxics. 2026 Aug 9;14(8):703. doi: 10.3390/toxics14080703 (PMC13517147; doi:10.3390/toxics14080703)
Supplement: Supplementary file 1 [file toxics-14-00703-s001.zip › toxics-4395543-supplementary.pdf]

SUPPORTING INFORMATION

Associations of Serum Substituted *p*-Phenylenediamine-Derived Quinone Levels with Oxidative Stress and Immune Function Biomarkers in a General Adult Population

Analysis of MDA and immune function biomarkers in human serum. Serum MDA levels were assayed via the thiobarbituric acid (TBA) reaction. In this method, MDA undergoes condensation with TBA under acidic conditions and elevated temperature, forming a stable pink-colored trimethine adduct. This chromogen exhibits maximum absorbance at 530 nm. Quantification was achieved spectrophotometrically using the Beer-Lambert law, where absorbance intensity is proportional to MDA concentration.

Serum concentrations of immunoglobulins (i.e., IgM, IgA, IgG) were determined by immunoturbidimetry. Each assay exploited specific antigen-antibody interactions. For IgM, it binds to anti-IgM antibodies, forming insoluble immune complexes. For IgA, it reacts with anti-IgA antibodies, generating antigen-antibody aggregates. For IgG, it forms complexes with anti-IgG antibodies in the reagent. In all cases, complex formation increased solution turbidity through light scattering. The change in turbidity was measured spectrophotometrically at 340 nm, with absorbance directly proportional to immunoglobulin concentration.

CRP levels were assessed using latex-enhanced immunoturbidimetry. Anti-CRP antibodies are covalently bound to latex particles. Upon addition of CRP, agglutination occurs as multiple CRP molecules cross-link the antibody-coated particles, forming large aggregates. This significantly amplifies turbidity. The aggregate density, quantified by absorbance at 562 nm, provides a sensitive measure of CRP concentration.

Table S1. List of target PPD-Qs in the present study and their abbreviations and full names.

Abbreviation	Full name
**IPPD-Q**	2-(isopropylamino)-5-(phenylamino)cyclohexa-2,5-diene-1,4-dione
**DPPD-Q**	2,5-bis(phenylamino)cyclohexa-2,5-diene-1,4-dione
**CPPD-Q**	2-(cyclohexylamino)-5-(phenylamino)cyclohexa-2,5-diene-1,4-dione
**6PPD-Q**	2-((4-methylpentan-2-yl)amino)-5-(phenylamino)cyclohexa-2,5-diene-1,4-dione
**77PD-Q**	2,5-bis((5-methylhexan-2-yl)amino)cyclohexa-2,5-diene-1,4-dione
**DTPD-Q**	2,5-bis(o-tolylamino)cyclohexa-2,5-diene-1,4-dione

Table S2. MRM transition parameters for PPD-Qs and internal standard.

	MRM transition		Cone voltage (eV)	Collision energy (eV)
	Parent ion	Daughter ion		
**6PPD-Q**	299	215	38	15
		187	38	27
**IPPD-Q**	257	187	33	28
		215	33	22
**DPPD-Q**	291	263	38	12
		170	38	30
**DTPD-Q**	319	301	40	14
		212	40	22
**CPPD-Q**	297	187	35	28
		98	35	35
**77PD-Q**	335	237	40	15
		139	40	28
**6PPD-Q-d_5**	304	192	38	27

Table S3. LODs and extraction recoveries of PPD-Qs in human serum.

		Extraction recovery (%)					
		Spiked at 0.5 ng/mL		Spiked at 5.0 ng/mL		Spiked at 15 ng/mL	
	LOD (ng/mL)	Mean	SD	Mean	SD	Mean	SD
**IPPD-Q**	0.025	97	7	96	7	83	13
**DPPD-Q**	0.098	101	13	84	7	94	8
**CPPD-Q**	0.036	102	9	107	8	88	10
**6PPD-Q**	0.072	90	14	87	14	105	4
**77PD-Q**	0.031	88	12	89	10	99	9
**DTPD-Q**	0.066	94	8	98	11	102	10

Table S4. Correlations in human serum concentrations among different PPD-Qs.

	Spearman's rho	CPPD-Q	77PD-Q	DPPD-Q	6PPD-Q	IPPD-Q
**DTPD-Q**	**Correlation Coefficient**	.192	.018	.201	.211	.464
	**Sig. (2-tailed)**	.307	.581	.506	.412	.040
**CPPD-Q**	**Correlation Coefficient**	1.000	.339	.109	.536	.357
	**Sig. (2-tailed)**	.	.000	.463	.000	.105
**77PD-Q**	**Correlation Coefficient**		1.000	.279	.417	.683
	**Sig. (2-tailed)**		.	.522	.028	.000
**DPPD-Q**	**Correlation Coefficient**			1.000	.530	.679
	**Sig. (2-tailed)**				.000	.000
**6PPD-Q**	**Correlation Coefficient**				1.000	.390
	**Sig. (2-tailed)**				.	.335

Table S5. Correlations between MDA and immune function biomarkers in human serum.

		IgA	IgG	IgM	MDA
**CRP**	**Spearman's Correlation**	.128	.477	-.056	-.531
	**Sig. (2-tailed)**	.405	.027	.697	.000
**IgA**	**Spearman's Correlation**	1	.519	.390	-.455
	**Sig. (2-tailed)**		.000	.031	.042
**IgG**	**Spearman's Correlation**		1	-.031	-.198
	**Sig. (2-tailed)**			.816	.215
**IgM**	**Spearman's Correlation**			1	.467
	**Sig. (2-tailed)**				.045
